# Evaluation of the impact of single nucleotide polymorphisms and primer mismatches on quantitative PCR

**DOI:** 10.1186/1472-6750-9-75

**Published:** 2009-08-28

**Authors:** Brian Boyle, Nancy Dallaire, John MacKay

**Affiliations:** 1 Centre d'Étude de la Forêt, Institut de biologie intégrative et des systèmes, Pav. C.E. Marchand, Université Laval, 1030 Rue de la Médecine, Quebec City (QC), G1V 0A6, Canada

## Abstract

**Background:**

Robust designs of PCR-based molecular diagnostic assays rely on the discrimination potential of sequence variants affecting primer-to-template annealing. However, for accurate quantitative PCR (qPCR) assessment of gene expression in populations with gene polymorphisms, the effects of sequence variants within primer binding sites must be minimized. This dichotomy in PCR applications prompted us to design experiments to specifically address the quantitative nature of PCR amplifications with oligonucleotides containing mismatches.

**Results:**

We performed qPCR reactions with several primer-target combinations and calculated ratios of molecules obtained with mismatch oligonucleotides to the average obtained with perfect match primer pairs. Amplifications were performed with genomic DNA and complementary DNA samples from different genotypes to validate the findings obtained with plasmid DNA. Our results demonstrate that PCR amplifications are driven by probabilities of oligonucleotides annealing to target sequences. Empiric probabilities can be measured for any primer pair. Alternatively, for primers containing mismatches, probabilities can be measured for individual primers and calculated for primer pairs.

**Conclusion:**

The ability to evaluate priming (and mispriming) rates and to predict their impacts provided a precise and quantitative description of assay performance. Priming probabilities were also found to be a good measure of analytical specificity.

## Background

Single nucleotide polymorphisms (SNPs) have posed a challenge to the study of gene expression because they affect methods based on oligonucleotide hybridization, such as microarrays and PCR. In a recent study, Walter and collaborators identified a large proportion of false positive and negative results when comparing two commonly used inbred mouse strains with Affimetrix microarrays [1]. Most of the discrepancies could be attributed to SNPs, which affected 16% of the probe sets. These results have highlighted the importance of considering SNPs during the design of hybridization-based assays such as microarrays and PCR, depending on their occurrence or frequency. Genomic diversity or the occurrence of sequence variants in genomes can be estimated by the comparison of any two identical chromosomes. In human genomes, a SNP is found in an individual every 1000–2000 bases, which constitutes a 0.1% per base rate of heterozygosity [2]. Moreover, the overall occurrence of SNPs increases with the addition of sequence information from new individuals or populations. The recent sequencing of a human genome from one individual, James D. Watson, by massively parallel DNA sequencing, identified 0.61 million new SNPs. Interestingly, 18% of the sequence variation found in Watson's genome was not present in dbSNP, the Single Nucleotide Polymorphism database [3]. Other studies have estimated similar rates of occurrence in the coding sequences of humans, *Drosophila *and plants, ranging from 0.4% to 2%, depending on the gene [4-7].

A further issue of concern for PCR-based assays is the frequency of SNPs within populations. Current estimates have predicted that a SNP with a population frequency of 1% occurs every 290 bases in the human genome [8]. Since most primer pairs used in qPCR span an average of 50 non-overlapping nucleotides, a significant proportion of SNPs (17% in humans) may be predicted to fall within primers based on chance alone. Consequently, the sensitivity of PCR-based gene expression assays to SNPs must be minimized for such assays to accurately measure transcript numbers in populations, especially in populations where genotypic variation has not been determined for the targeted genes. In a related issue, the detection of multiple sequence variants (strains) of a pathogen is particularly important in molecular diagnostics for viral quantification assays where considerable sequence variation between strains and subtypes can be observed [9].

Besides issues regarding laboratory setup [10], PCR false positives also can arise when amplification is detected as a result of mispriming. The term "mispriming" can be used when PCR products are generated through primer annealing to partially complementary sequences. Mispriming is also of great concern in clinical molecular diagnostics, especially when the PCR assay must discriminate between closely related sequences [11,12]. It could become even more important when the targeted sequence is diluted in a pool of closely related interfering sequences. Therefore, the challenge in developing PCR-based molecular diagnostic assays is the degree of certainty with which assays may be able to detect all of the molecules of interest without detecting interfering molecules.

Sensitivity or the lack of sensitivity to sequence variants represents an important dichotomy in PCR applications. Consequently, assay performance with respect to target and non-target molecules must be precisely described to facilitate assay selection and/or validation. For the current study, we designed experiments to specifically address the quantitative nature of mispriming caused by sequence variants. We demonstrate that PCR priming occurs with a measurable frequency and it could be used as a mean of quantitatively describing and evaluating PCR assay performance. Experimentation was performed on genomic DNA (gDNA) and complementary DNA (cDNA) to support these findings, and on plasmid DNA to evaluate the impact of PCR parameters on priming frequency.

## Methods

### ESTs (Expressed Sequence Tags), clones, sequence alignments and primer design

*Lim1 *ESTs used in this study were from previously described cDNA libraries [13] and have been identified in ForestTreeDB [14]. Clones are available through the Arborea Project website  and Arborea EST sequences are deposited in GenBank [GenBank: DV975691, GenBank:DV977042, GenBank:DV976393, GenBank:DV977754, GenBank:DV977683, and GenBank:DV976321].

Sequence alignments (See Additional file 1) were performed with the BioEdit biological sequence alignment editor, which is freely available on the web at the following address 

**Figure 1:**
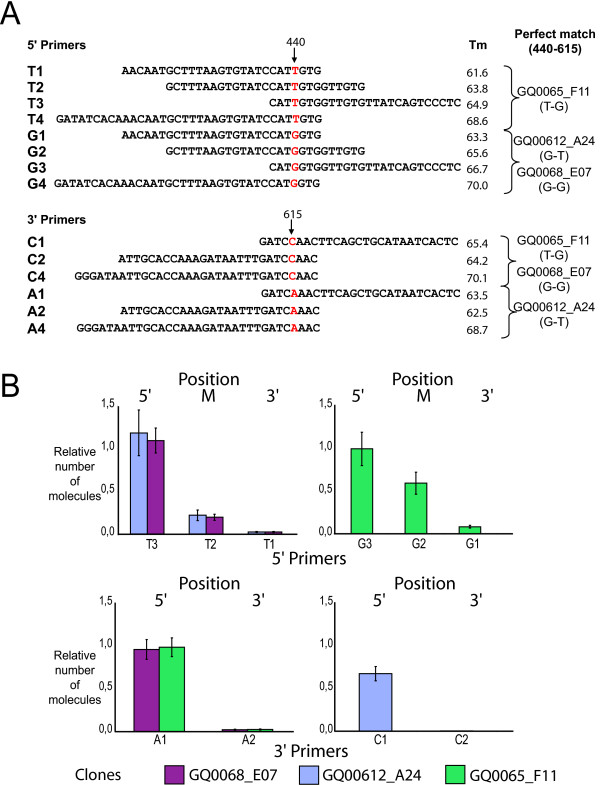
**Single nucleotide polymorphisms (SNPs) affect real time PCR quantifications**. (A) Oligonucleotides used in this study. The positions of SNPs are indicated by arrows. Perfect match cDNA clones are identified on the right with the nucleotides SNPs at position 440 and 615 indicated in parentheses. Tm: melting temperature calculated with Primer3 software. (B) Positional effect of the SNPs. The position of the SNP within the oligonucleotide is indicated: 5', middle (M) or 3'. Relative numbers of molecules represent the ratio of molecules detected with mismatch primers relative to the number of molecules detected with perfect match primer pairs.

The software Primer3 [15] was used to validate primer selection and to perform Tm (melting temperature) calculations. Primer sequences are shown in Figure 1.

### Plant material, DNA and RNA extractions

Plant material was taken from 37-year-old trees in a progeny trial of white spruce (*Picea glauca *(Moench) Voss) that had been established near Quebec City (QC, Canada). The trial was composed of 40 half-sib families (obtained by wind-pollination), which originated from different areas of Eastern Canada. Three tissue samples were collected from the main stem of each tree at 1.5 m above ground level using a 16 mm leather punch. A 1-mm thick sample of actively growing tissue was taken from either side of the cambial zone, and represented secondary phloem and xylem. The samples were immediately frozen in liquid nitrogen. Frozen material was ground to fine powder using liquid nitrogen-cooled 50 ml jars/25 mm beads in a ball mill (MM300 Mixer Mill, Retsch GmbH, Haan, Germany). DNA extractions were performed on 100 mg of liquid N_2_-ground secondary phloem using DNeasy Plant Mini kit (QIAGEN, Germantown, MD, USA) according to the manufacturer's instructions. RNA extractions were performed as previously described for white spruce [16]. RNA integrity was checked with a 2100 Bioanalyzer (Agilent Technologies, Santa Clara, CA, USA).

### Genotyping

In order to determine the genotype of each tree, a portion of the *Lim1 *gene was amplified from trees using forward (ACCAGTATGCCTTCATTGTGTTC) and reverse primers (AAAGACCAATGTCCCTAATAGTCATG). The resulting PCR fragments were sequenced using the same primers that were used to genotype the individuals at the Plate-forme d'analyses biomoléculaires (Université Laval, Quebec City, QC, Canada). The presence of double peaks in the sequencing reaction chromatogram identified the heterozygote individuals.

### cDNA preparation and quantitative PCR

Complementary DNAs were prepared from 500 ng of total RNA using the first strand cDNA synthesis system (Invitrogen, Carlsbad, CA, USA). PCR mixtures that contained QuantiTect SYBR Green PCR kit (QIAGEN), QuantiFast SYBR Green PCR kit (QIAGEN) or LightCycler 480 SYBR Green I Master (Roche, Basel, Switzerland) were as follows: 1× master mix, 300 nM of 5' and 3' primers, and target DNA or cDNA (10 ng) in a final volume of 15 μl. Reactions were setup using an epMotion 5075 pipetting robot (Eppendorf, Hamburg, Germany) and amplifications were carried out in a LightCycler 480 (Roche). Cycling for both QuantiTect and QuantiFast mixtures was performed as follows: an initial 15-min activation step at 95°C, followed by 50 cycles of 94°C for 10 s and 62°C for 2 min (QuantiTect) or 1 min (QuantiFast); a single fluorescent read was taken after each cycle immediately following the annealing and elongation step at 62°C. Three-step cycling (50 cycles) was performed for the Roche master mix, according to instructions provided by the manufacturer. Melting curve analysis was performed at the end of cycling to ensure single product amplification of the appropriate melting temperature. Experiment description and data presentation follow the guidelines on the minimal information for publication of quantitative PCR experiments (MIQE) [17].

### Determination of the number of molecules (LRE method)

The methodology described here is a slight modification of the procedure elaborated by Rutledge and Stewart [18]. Insertion of equation (2) into equation (1), both described in Rutledge and Stewart, served to derive a new equation (3) that was used to quantify molecules.

(1)

(2)

(3)

In these equations, F_0 _is the initial target quantity expressed in fluorescence units, F_max _is the maximal fluorescence reached at the plateau phase where the efficiency of the PCR reaction reaches 0, E_max _is the maximal efficiency that occurs at the beginning of cycling, C_1/2 _is the reaction cycle located at the inflection point of the fluorescence curve where the fluorescence is half of F_max _and the efficiency is half of E_max_, and ΔE represents the rate of loss in efficiency. For each amplification reaction, ΔE and E_max _were determined using the linear regression of efficiency (LRE) method [18] and C_1/2 _was calculated by taking the first derivative of the fluorescent readings. F_0 _values were then transformed to molecules (N_0_) with equations described in Rutledge and Stewart [18]. Fluorescence background was removed prior to LRE analysis and C_1/2 _determination. Optical calibrations of the master mixes in the LC480 were performed with lambda DNA as previously described [18]. An Excel spreadsheet designed to accommodate the 384 sample output from the LC480 was created to automatically convert fluorescent reads to molecules. Excel formulas, macros and tutorial are available from the Arborea website publication section .

## Results

To evaluate the effect of sequence variants on quantification by qPCR, we designed primers against polymorphic sites within the coding sequence of a single gene in white spruce (Figure 1A). This gene was represented by 6 cDNA clones (12 reads) in our EST database [13], and three SNPs were identified within a 700 bp region of the EST sequences (i.e., 1 every 233 bp). Two of the SNPs were located 165 bp apart and were represented in three different alleles (clones), which constituted ideal templates for studying the impact of SNPs on qPCR (see Additional file 1 for the sequence alignment). Amplifications were performed with these three cDNA clones and 24 primer pairs designed to have melting temperatures between 62–67°C. Seven concentrations of each plasmid were used with all 24 primer pairs; reactions were run in duplicate wells and in duplicate runs (4 quantifications for each plasmid/primer pair/concentration). Each clone had 6 perfect match primer pairs and 18 mismatch combinations (a mismatch in one or two primers).

### Conversion of raw fluorescence data to molecules

**Figure 2:**
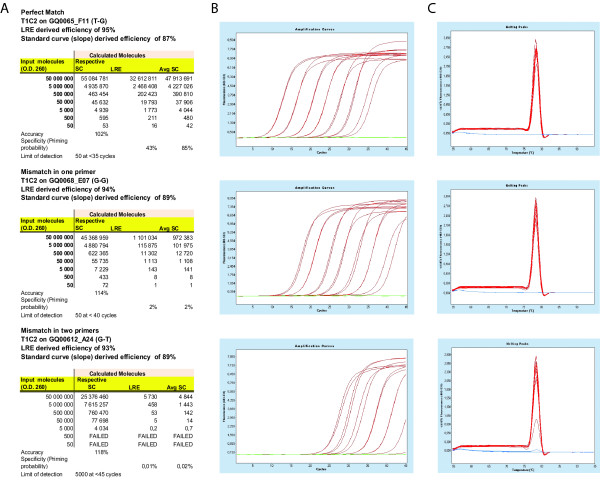
**Amplification profiles and determination of the number molecules with perfect match and mismatch primers**. (A) Data generated from amplification profiles presented in (B). The number of molecules calculated with different methods is presented. The respective standard curve (SC) data is directly linked to the amplification profiles shown in (B) and input number of molecules. The LRE method is dependent only on fluorescence data (B) and instrument calibration. The Avg SC data is derived from an average standard curves obtained with perfect match primer pairs. Accuracy is calculated with the respective standard curves and represents the average ratio of observed to input molecules. Specificity is calculated with LRE or Avg SC data and represents the average ratio of observed to input molecules. Sensitivity is the limit of detection of an assay and is defined as the lowest input number of molecules generating a complete amplification profile [17]. The input number of molecules was determined optically using a spectrophotometer. (C) Melting profiles associated to the amplification profiles presented in (B).

**Figure 3:**
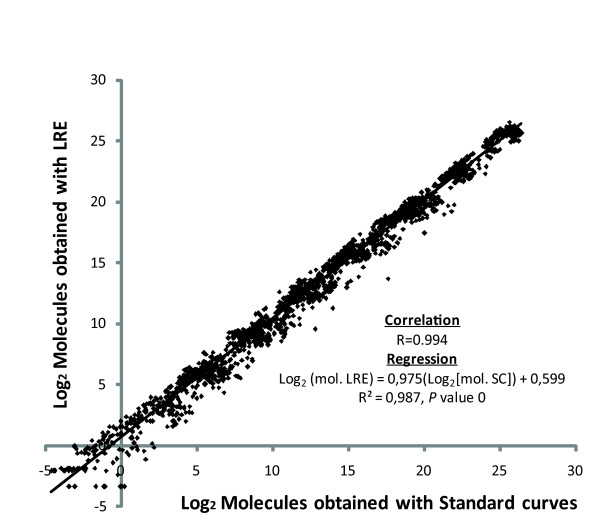
**Relationship between the number of molecules calculated with LRE and average standard curves methods**. The strong correlation (R of 0.994) indicates that both methods generate similar data. The slope of the linear regression being close to 1 and the intercept near 0 indicate that the numbers of molecules reported by each technology are almost identical. This is supported by an R^2 ^of 0.987 and an excellent *P *value of 0.0.

To quantitatively assess the impact of primer mismatches, the qPCR data must be converted to numbers of molecules. Two methods were used to convert the raw fluorescence data to molecules: (1) the commonly used method based on quantification cycle (C_q_) values and standard curves, and (2) the linear regression of efficiency method (LRE) developed by Rutledge and Stewart (2008). Amplifications from serial dilutions of target molecules were used to build standard curves (see Additional file 1). The standard curves were then used to convert C_q _values to molecules. Standard curves depend upon two things: the positioning of the amplification profiles (C_q_) and the input number of molecules. As a consequence, standard curves always reported numbers of molecules based on the input number of molecules, even when they were based on profiles that are shifted relative to perfect match amplifications (Figure 2). Therefore, average standard curves had to be built with perfect match primer pairs (see Additional file 1) and used to convert C_q _values to molecules. This was possible only because the slope-derived primer pair efficiency of nearly all primer pairs was in the range of 85 to 90% indicating that primer pairs worked well with any of the given targets and because of the low variability of C_q _values obtained with perfect match primer pairs (see Additional file 1). Alternatively, the LRE method, derived from sigmoidal modeling, uses raw fluorescence data to calculate molecules and amplification efficiency for each sample, and thus has the advantage of not requiring or relying on standard curves. There was a high correlation (R of 0.994) between the numbers of molecules calculated with LRE and average standard curves (Figure 3). Since LRE and average standard curves quantification methods generated highly correlated results, LRE was used for further data presentation.

Coefficients of variation (CVs) calculated on molecule numbers for each of the primer pairs on a single template (intra-assay variation) were between 14 and 25% (see Additional file 1) which are comparable to intra-assay variations observed elsewhere [19-21]. The CVs calculated on molecules among replicates of all amplifications with perfect match primer pairs (combination of intra- and inter-assay variation) did not exceed 40% (CVs usually between 20–30%), except in the case of low plasmid concentrations (see Additional file 1). On the other hand, the variances (VAR) were proportional to the numbers of molecules detected (see Additional file 1); therefore, more abundant targets gave larger SD and VAR than less abundant targets, even though CVs were uniform. A log_2 _transformation was applied to the number of molecules for statistical analyses to have more uniform variances (see Additional file 1). The robustness of our approach in evaluating the impact of SNPs was indicated by the low degree of variability of log_2 _transformed data obtained by perfect match primer pairs and by the close match to the number of molecules determined optically at 260 nm (see Additional file 1).

### Measuring mispriming frequency

This experimental setup was used to evaluate the impact of SNPs on amplification efficiency. Results showed that SNPs did not have a significant impact on efficiency since perfect match and mismatch primer pairs had nearly identical amplification efficiencies (see Additional file 1). This implies that once the mismatch amplifications had occurred, the amplification profiles were identical to profiles produced by perfect match primer pairs. It also suggested that primer annealing to non-target molecules was independent of components that drive PCR amplification efficiencies. However, amplification efficiency is largely influenced by primer annealing to target molecules and it was entirely possible that additives favoring annealing directly influenced amplification efficiency and vice versa.

In contrast, the presence of a single mismatch in one of the primers significantly decreased the number of molecules detected when compared to pairs of perfect match primers, as might be expected (Table 1 and see Additional file 1). When using a perfect match primer pair, the number of molecules amplified in the first cycle was expected to be a function of the amplification efficiency. Since amplification efficiency was the same when using perfect match and mismatch primer pairs, the most likely explanation for the differences in the number of molecules resides in the capacity for mismatch amplifications. We used our experimental setup to investigate the parameters that govern quantification with mismatch primers. First, a ratio (log_2 _differences) was calculated based on the number of molecules measured with primers containing mismatches (one mismatch in one primer or in both primers) over that obtained with perfect match primer pairs (Table 1). We observed nearly identical ratios for all seven dilutions of a given target, ranging from 50,000,000 to 50 molecules, when the same mismatch primer pair was used (see Additional file 1). The consistency with which these ratios, or relative frequencies, are observed suggests that they are empirical probabilities of mismatch amplification (mispriming) in the first cycle or first few cycles. This is consistent with the number of molecules present in most amplifications being several magnitudes over the measured probabilities and it takes into account that mismatch priming generates the same number of molecules at each cycle which are perfect match templates amplified exponentially in following cycles.

**Table 1:** Ratio of the observed number of molecules with mismatch primers relative to the average number of molecules with perfect match primer pairs.

	**QIAGEN**		**ROCHE**	
**Primer Pairs^1 ^(5' – 3')**	**Observed Effect^2^**	**Predicted Effect^3^**	**Observed Effect^2^**	**Predicted Effect^3^**
**Clone GQ0068_E07 (G-G)**				
**T1 – PM**	0.021		0.40	
**T2 – PM**	0.20		0.79	
**T3 – PM**	1.2		0.94	
**PM – A1**	1.0		1.2	
**PM – A2**	0.019		0.52	
**T1-A1**	0.022	0.021	0.49	0.47
**T1-A2**	0.00047	0.00039	0.14	0.21
**T2-A1**	0.24	0.21	1.1	0.93
**T2-A2**	0.0045	0.0037	0.35	0.41
**T3-A1**	1.3	1.2	1.4	1.1
**T3-A2**	0.025	0.022	0.44	0.49

**Clone GQ0065_F11 (T-G)**				
**G1 – PM**	0.089		0.79	
**G2 – PM**	0.68		1.3	
**G3 – PM**	1.1		1.1	
**PM – A1**	1.1		1.1	
**PM – A2**	0.024		0.45	
**G1-A1**	0.10	0.098	1.1	0.90
**G1-A2**	0.0024	0.0021	0.56	0.35
**G2-A1**	0.66	0.74	1.4	1.4
**G2-A2**	0.013	0.016	0.58	0.56
**G3-A1**	1.0	1.2	1.5	1.2
**G3-A2**	0.022	0.027	0.64	0.48

**Clone GQ00612_A24 (G-T)**				
**T1 – PM**	0.023		n.d.^4^	
**T2 – PM**	0.20		n.d.	
**T3 – PM**	1.2		n.d.	
**PM – C1**	0.87		n.d.	
**PM – C2**	0.0062		n.d.	
**T1-C1**	0.018	0.020	n.d.	
**T1-C2**	0.00012	0.00014	n.d.	
**T2-C1**	0.15	0.17	n.d.	
**T2-C2**	0.0015	0.0012	n.d.	
**T3-C1**	0.95	1.1	n.d.	
**T3-C2**	0.0075	0.0076	n.d.	

^1 ^Primer pairs with one mismatch primer containing in a single oligonucleotide substitution in combination with one perfect match primer (PM) are used to evaluate the effect of SNP (observed effect) on qPCR quantifications.

^2 ^Observed effects are expressed as a ratio of quantified molecules relative to the number of molecules quantified with perfect match primer pairs.

^3 ^Predicted effects are calculated when two mismatch primers are used, by multiplying the observed effect of each of the two single mismatch primers. For example the predicted effect of the T1-A1 (0.021) primer pair on clone GQ0068_E07 is equal to the product of the observed effects of T1 (T1-PM, 0.021) and A1 (PM-A1, 1.0). There is strong agreement between the observed and predicted effects.

^4 ^not determined

Since mismatches in primers cause a shift in amplification profiles (Figure 2) we calculated the differences between quantification cycles (C_q_) generated by reactions containing primers with mismatches to the average C_q _obtained with perfect match primers (ΔC_q _method; see Additional file 1). As expected, the results are highly concordant with those obtained with log_2 _ratios (Table 2). The opposite signs of the LRE based Log_2 _ratios and ΔC_q _are inherent to units used with each method: the smaller number of molecules with mismatch primer pairs gives a decreased log_2 _number of molecules and a higher C_q _than the average with perfect match primers. Probabilities are equivalent to 2^(log2ratio) ^or E^-ΔCq^, where E is equivalent to the primer pair efficiency (between 1,84 and 1,96 depending on the primer pair and the method selected to estimate efficiency; see Additional file 1).

**Table 2:** Comparison of the results obtained with LRE based log_2 _ratios to the ΔC_q _analysis.

	**LRE based analysis**			**ΔC_q _based analysis**		
**Mismatch Primer Pair**	**Log_2 _Ratio**	**SD**	***P *Value^1^**	**ΔC_q_**	**SD**	***P *Value^1^**

**Clone GQ00612 _A24 (G-T)**						
T1-PM	-5,4	0,6	**3,4E-141**	5,9	1,0	**4,9E-109**
T2-PM	-2,3	0,7	**1,3E-66**	2,9	1,0	**1,3E-54**
T3-PM	0,3	0,5	1,4E-02	0,0	0,9	2,7E+01
PM-C1	-0,2	0,4	2,1E-03	0,1	0,3	1,4E+01
PM-C2	-7,3	0,6	**7,1E-195**	6,9	0,4	**1,4E-155**
T1-C1	-5,8	0,6	**7,8E-113**	6,0	0,7	**4,0E-84**
T1-C2	-13,1	0,9	**2,4E-156**	13,2	0,7	**7,1E-130**
T2-C1	-2,7	0,6	**2,0E-59**	3,0	0,5	**1,3E-43**
T2-C2	-9,4	0,6	**2,1E-136**	9,6	0,9	**4,9E-108**
T3-C1	-0,1	0,5	5,5E-01	0,2	0,4	1,0E+01
T3-C2	-7,1	0,5	**2,3E-99**	7,0	0,6	**1,4E-79**

**Clone GQ0068 _E07 (G-G)**						
T1-PM	-5,6	0,8	**6,7E-154**	6,0	0,7	**1,0E-164**
T2-PM	-2,3	0,4	**3,9E-97**	2,8	0,4	**4,2E-123**
T3-PM	0,2	0,3	2,2E-04	0,0	0,4	2,9E+01
PM-A1	0,0	0,3	1,8E-02	0,2	0,3	2,3E-03
PM-A2	-5,8	0,8	**2,3E-173**	6,7	0,6	**1,1E-211**
T1-A1	-5,5	0,6	**1,6E-126**	6,2	0,7	**3,9E-141**
T1-A2	-11,1	0,8	**1,2E-171**	12,5	0,7	**8,3E-186**
T2-A1	-2,1	0,5	**1,4E-59**	2,8	0,6	**3,6E-85**
T2-A2	-7,8	0,6	**1,7E-157**	9,4	0,5	**9,3E-178**
T3-A1	0,4	0,4	2,0E-04	0,0	0,3	3,2E+01
T3-A2	-5,3	0,9	**1,1E-120**	6,6	0,7	**1,2E-144**

**Clone GQ0065_F11 (T-G)**						
G1-PM	-3,5	0,7	**1,8E-109**	3,5	0,7	**3,9E-113**
G2-PM	-0,6	0,5	**6,3E-15**	0,4	0,5	**2,1E-08**
G3-PM	0,1	0,4	8,6E-04	-0,2	0,3	5,7E+00
PM-A1	0,1	0,4	6,4E-04	0,0	0,4	2,9E+01
PM-A2	-5,4	0,7	**1,6E-164**	6,2	0,6	**1,5E-191**
G1-A1	-3,3	0,5	**4,4E-75**	3,5	0,5	**1,5E-94**
G1-A2	-8,7	0,7	**2,7E-152**	9,5	0,9	**1,2E-157**
G2-A1	-0,6	0,5	**1,7E-09**	0,6	0,5	**2,5E-09**
G2-A2	-6,3	0,7	**2,3E-129**	6,7	0,4	**4,6E-147**
G3-A1	0,0	0,3	6,1E-01	0,1	0,3	1,7E+01
G3-A2	-5,5	0,6	**1,6E-117**	6,0	0,5	**1,3E-138**

^1 ^*P *values are the result of a two tailed homoscedastic Student T-test comparing log_2 _ratios or ΔC_q _obtained with mismatch primers pairs to log_2 _ratios or ΔC_q _obtained with perfect match primer pairs (a Bonferroni correction for multiple testing was applied). The values in bold are very highly significant (*P *< 10^-4^).

### Predicting the impact of SNPs in both primers

**Figure 4:**
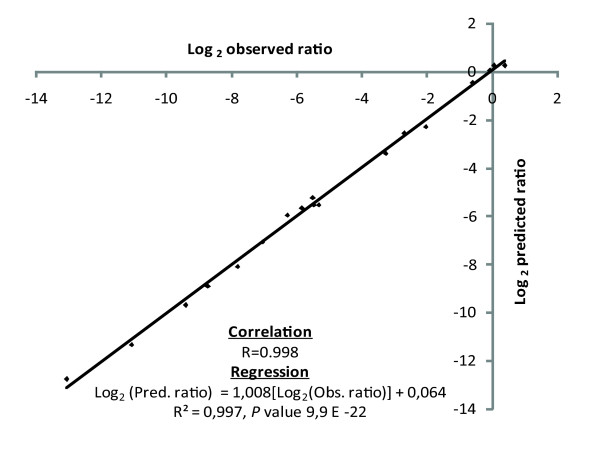
**Relationship between predicted and observed number of molecules**. The strong correlation (R = 0.998) indicates that the rate of PCR mispriming of a primer pair can be predicted based on the mispriming probability measured for individual primers.

We hypothesized that the probability for a misprimed amplification was independent for each oligonucleotide in the reaction; therefore, it should be possible to predict the results obtained with a mismatch in both of the oligonucleotides, based on the ratios obtained for each mismatch primer used with a perfect match primer. If such was the case, the probability of amplification should be equal to the product of their probabilities, or the sum of their log_2 _probabilities, according to the multiplication rule of probability [22]. For example, the predicted probability of T1-A2 amplifying (p: 0.0005, log_2_p: -11.06) from clone GQ0068_E07 should be equal to the product of probabilities (sum of log_2 _probabilities) associated with T1 (p T1-PM: 0.0222, log_2_p: -5.56) and A2 (p PM-A2: 0.0204, log_2_p: -5.75) (Table 1, see Additional file 1). This hypothesis was validated by the very high correlation between the predicted and observed effects for each of 18 primer pairs containing a mismatch in both oligonucleotides, and was consistent with two different PCR master mixes (Figure 4). It also validated nicely with the ΔC_q _method (see Additional file 1). This capacity to predict held for all concentrations tested including amplification failures when the number of molecules in the sample fell below the probability of amplification (see Additional file 1). In other words, it was possible to determine an empirical probability of mismatch amplification of an oligonucleotide and calculate the probability of mispriming of a primer pair for a given target under controlled PCR conditions.

Two other findings related to PCR attracted our attention. First, the position of the SNP within the oligonucleotides influenced the probability of mismatch amplification, i.e., a SNP located at the 3' end influenced quantification more strongly than the same SNP located at the 5' end of the primer (Figure 1B, Table 2). This finding was not surprising since this principle is routinely used to increase specificity in PCR assay design. Second, the same set of primers was tested at the same temperature, but with a different qPCR master mix. Although the same conclusions can be drawn, the results obtained with the second mix were less affected by mismatches (Table 1). This second finding was less expected since PCR specificity is thought to mainly reside in primer design and annealing temperatures. This observation is meaningful because it indicated that priming probabilities may be used to assess very different PCR assays.

### Using priming probabilities to describe assay performance

These observations lead us to predict that priming probabilities could be used to predict the performance of two assays, if they are inherent characteristics of PCR assays. We compared two assays that used the same oligonucleotides designed to detect each allele identified from our EST database (see Figure 1) and the same annealing and elongation (A&E) temperature. The difference between the two assays resided in the use of master mixes, which were provided by the same manufacturer (QuantiTect or QuantiFast from QIAGEN), and in the A&E time (1 min vs. 2 min). Priming, or mispriming, probabilities can predict large differences in assay specificity, or sensitivity to SNPs, even though they use the same oligonucleotides at the same A&E temperature; here, the assay with a shorter A&E time was predicted to have less specificity (Table 3). Based on priming probabilities, we predicted that Assay 1 had the greater potential for discriminating between targets and closely interfering molecules. Its priming probabilities for perfect match targets varied between 0.70 to 1.0, whereas the probabilities for non-targets varied from 0.085 to 0.0002, which represented discrimination potentials ranging from more than ten-fold to several thousand-fold, respectively. The capacity of Assay 2 to discriminate between targets and closely interfering sequences, i.e., alleles in this particular case, was predicted to be weak because the priming (mispriming) probabilities for non-targets were between 0.65 and 1.2. This meant that most non-target molecules were amplified efficiently compared to target molecules, which had priming probabilities between 0.91 and 1.1.

**Table 3:** PCR assay properties.

**Properties**	**Assay 1**			**Assay 2**		
**Master mix**	Quantitect			Quantifast		
**A&E temperature**	62°C			62°C		
**A&E time**	2 min			1 min		
**Priming probabilities**						
						
	99% Confidence interval^1^			99% Confidence interval^1^		
	Lower	Mean	Upper	Lower	Mean	Upper
T1-C2 on T-G (PM)	0.39	0.79	1.6	0.48	1.1	2.3
G1-C2 on G-G (PM)	0.49	1.0	2.1	0.50	0.95	1.8
G1-A2 on G-T (PM)	0.31	0.70	1.5	0.52	0.91	1.5
T1-C2 on G-T (2)	0.0000032	0.00021	0.0011	0.21	0.51	1.2
T1-C2 on G-G (1)	0.0090	0.015	0.025	0.41	1.2	3.5
G1-C2 on T-G (1)	0.048	0.085	0.15	0.34	0.79	1.8
G1-C2 on G-T (1)	0.0019	0.0061	0.019	0.28	0.65	1.5
G1-A2 on T-G (2)	0.00034	0.0030	0.025	0.25	0.83	2.7
G1-A2 on G-G (1)	0.0026	0.020	0.16	0.51	1.1	2.4

^1^Three standard deviations above (upper) or below (lower) the mean. Means and intervals were calculated using log_2 _transformed data.

**Figure 5:**
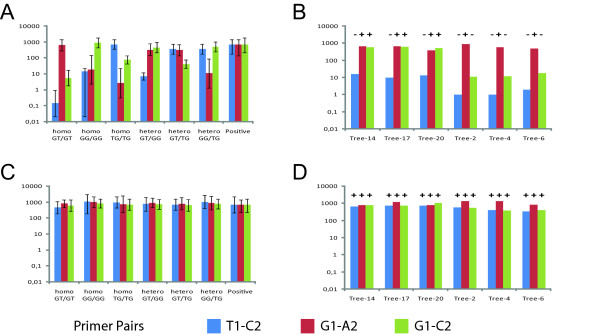
**Assay comparison for genotyping**. The discrimination potential of Assay1 (A, B) and Assay2 (C, D) was evaluated with 10 ng of genomic DNA. Panels A and C represent the number of molecules predicted for known genotypes according to the priming probabilities presented in Table 3. The error bars in panels A and C are 99% confidence intervals associated with each value. Panels B and D are the results obtained from individual trees of unknown genotypes. The (+) and (-) above each bar indicate whether the observed number of molecules is within (+) or outside (-) of the 99% confidence interval from panels A and C, respectively. This criterion was used to discriminate between the presence or absence of an allele. Thus, comparison of panels A and C enables genotyping with Assay1: the numbers of molecules for Trees 14, 17, 20 are within the predicted intervals for a GT/GG heterozygote. Similarly, the numbers of molecules for Trees 2, 4, 6 are within the intervals predicted for a GT/GT homozygote. In contrast, the numbers of molecules predicted for all genotypes with Assay2 are too similar to one another to assign genotypes.

In order to validate our predictions based on priming probabilities, we performed genotyping assays on genomic DNA from individual white spruce trees (which have a diploid genome) to verify the allele discrimination potential of each assay. The expected number of molecules was calculated for 10 ng of genomic DNA for all homozygous and heterozygous genotypes at these SNP sites (Figure 5). We estimated the number of molecules expected from 10 ng of white spruce gDNA to be 912 since the genome size has been estimated to be around 20 Gb. Consequently, the number of molecules expected from each allele was equal to its priming probability (Table 3) multiplied by 456, and the sum of all alleles represents the number of molecules expected from an individual (Figure 5A, C). The genotype of individuals could thus be inferred by comparing the predicted and observed number of molecules. Rapid examination of predicted values of both assays (Figure 5A, C) clearly illustrates the better discrimination potential of Assay1 in comparison with Assay2. Consequently, it was possible to genotype individuals using Assay1 (Figure 5A, B), whereas this task was impossible with Assay2 (Figure 5C, D). We used 99% confidence intervals to score for the presence or absence alleles. This analysis correctly identified the presence of each allele in all individuals when using Assay1 conditions (Figure 5). The predicted genotypes of the 3 G-T/G-T homozygote (Trees 2, 4, 6) and the 3 G-T/G-G heterozygote (Trees 14, 17, 20) individuals identified with Assay1 were confirmed by amplifying and sequencing the genomic region containing the SNPs (see methods). The use of Assay 2 conditions was inefficient at discriminating between alleles even though the intervals of confidences of Assay2 were similar to Assay1 (Table 3), and gave positive scores for all three alleles in every one of these diploid samples.

These same two assays were also compared for determining RNA transcript levels among trees from the two genotypes previously determined (Figure 5-B) with perfect match and mismatch primers. Assay 2 gave less than a two-fold difference between the average transcript levels for the two groups of individuals, regardless of the pair of primers that was used (Table 4). This result was within the variation acceptable for a reference gene, particularly on non-normalized data. This observation indicates that Assay 2 which lacks specificity and has a reduced capacity to discriminate between alleles is better suited for determining transcript levels in a population of genetically variable individuals. In contrast, Assay1 conditions gave up to a 30-fold difference in transcript number when the primer was designed based on the G-G allele alone (SNP not accounted for) (Table 4). These differences were of similar magnitude than those observed with genomic DNA for these same trees. In the case of gene expression studies, Assay 1 conditions might lead to spurious results if a single primer pair was used on non-genotyped individuals.

**Table 4:** Validation of predicted assay performance on cDNA (Molecules measured from the cDNA equivalent of 10 nanograms of total RNA from secondary phloem).

	**Assay 1** **(Quantitect, 2 min A&E)** **Primer Pairs**			**Assay 2** **(Quantifast, 1 min A&E)** **Primer Pairs**		
**Tree number (genotype)**	**T1-C2**	**G1-A2**	**G1-C2**	**T1-C2**	**G1-A2**	**G1-C2**
Tree-14 (G-T/G-G)	1100	16000	18000	17000	53000	47000
Tree-17 (G-T/G-G)	1400	23000	29000	22000	54000	31000
Tree -20 (G-T/G-G)	1500	32000	21000	30000	81000	74000
**Mean ± SD**	**1400 ± 200**	**24000 ± 8000**	**23000 ± 6000**	**23000 ± 7000**	**63000 ± 16000**	**51000 ± 22000**
Tree-2 (G-T/G-T)	18	24000	380	11000	27000	20000
Tree-4 (G-T/G-T)	67	51000	1100	15000	35000	25000
Tree-6 (G-T/G-T)	69	54000	630	23000	60000	47000
**Mean ± SD**	**51 ± 29**	**43000 ± 16000**	**690 ± 30**	**17000 ± 6000**	**41000 ± 17000**	**31000 ± 14000**

Results presented in Figure 5 and Table 4 confirmed the prediction based on priming probabilities that Assay 1 has greater discriminatory power than Assay 2. Better discrimination is useful for genotyping assays or molecular diagnostics, whereas less sensitivity to SNPs is useful to buffer the effects of genotypic variation in gene expression assays. These results show that the use of priming probabilities provided a precise and quantitative description of assay performance, for two assays which had not previously been tested or optimized for diagnostic or gene expression purposes.

### Factors influencing primer to template annealing

Our results indicated that PCR parameters greatly influence the rate of primer annealing to target molecules. Therefore, we explored the potential impact of modifying PCR conditions on priming probabilities during qPCR quantifications with test primers containing a mismatch near the 3' end. We tested parameters that influence primer template annealing and elongation. Increasing the primer Tm or reducing the annealing temperature reduced the impact of SNPs (see Additional file 1). Changing both parameters simultaneously resulted in further attenuation. No further increase in the number of molecules were observed once all molecules were quantified, and the ratio of observed to expected molecules stabilized around 1. Increasing the annealing and elongation time also reduced the impact of SNPs (see Additional file 1). However, low plasmid concentrations occasionally gave abnormally large numbers of molecules (ratio of observed to expected molecules above 1.5) with the fast mixes (Roche and QuantiFast). This was particularly true with higher Tm oligonucleotides. Since no artefactual melting profiles were observed, we concluded that this problem may be linked to the additives introduced in fast mixes to favor primer template annealing which may cause repriming within a single round of amplification, under certain conditions.

Generally, the impact of SNPs was reduced by conditions that favor primer to template annealing, including lower cycling temperature relative to primer Tm, and longer annealing and elongation times. Both of the "fast" master mixes that were tested (Roche and QuantiFast) were less impacted by SNPs than QuantiTect (Table 1, see Additional file 1).

## Discussion

This study provided evidence that PCR mispriming (or priming) occurs with measurable frequencies relative to the expected number of molecules. Consequently, such frequencies can be considered as empirical probabilities. We demonstrated that the probability of generating an amplicon is equal to the product of the annealing probabilities of the individual primers as expected from two events with independent probabilities.

### Validation of the LRE methodology

To date, there is no consensus method to perform qPCR data analysis. The LRE procedure is simple to use once formulas are programmed (for example, in an Excel worksheet) and the qPCR apparatus has been calibrated. The raw fluorescence data is exported from the qPCR instrument to directly calculate numbers of molecules and amplification efficiencies from individual reactions (wells). In contrast, for the construction of standard curves, quantification cycle (C_q_) values are determined using the second derivative maximum in the LC480 software supplied with the instrument. These C_q _values obtained with serial dilutions of target molecules are used to derive standard curves which were in turn used to convert C_q _values to molecules. Standard curves are generated for each primer pair against each target. Primer pair efficiencies are derived from the slope of the linear regression of the standard curves. There are several standard curves when dealing with primer mismatches; therefore, a problem arises as to which standard curve to use. Primer mismatches cause shifts in the fluorescence profiles resulting in higher C_q _values (Figure 2). This shift to higher C_q _values caused by primer mismatches should have been indicative of lower number of molecules. However, converting back C_q _values to molecules using the respective standard curves always reported the number of molecules determined optically at 260 nm indicating that standard curves have the capacity to compensate for primer mismatches. To avoid this problem we constructed average standard curves with perfect match primers and used these as reference to evaluate the impact of mismatches. Results were nearly identical when using the average standard curve approach and the LRE method, clearly indicating that LRE is a valid methodology for analyzing qPCR data as previously reported [18]. However, although very highly correlated, the numbers of molecules determined by the LRE method were slightly different from the input numbers of molecules and average standard curve data (ratio to input molecules of 0.69, 0.73 and 1.27; see Additional file 1). These differences could be most likely attributed to the different manipulations required to produce serial dilutions of plasmid DNA (plasmid DNA isolation, restriction, optical assessment of DNA concentration and serial dilutions). In other words, the standard curve methods were based on input numbers of molecules added to the reaction, whereas the LRE determined molecules were measured from the reaction. This slight discrepancy between LRE and standard curve data, or input numbers of molecules, should not be considered a limitation to the study.

Since variances increase along with molecule numbers in qPCR (heteroscedasticity), we used a log_2 _transformation for statistical analyses of qPCR data. Although log transformation has been proposed earlier, log_2 _may be more representative of PCR reactions as molecules nearly double at each cycle. We found that log_2 _transformations generate variances similar to the ones observed with C_q _determination (see Additional file 1).

### Mispriming probabilities and molecular diagnostic

The ability to assign a rate of success or failure to a given assay has tremendous implications in PCR-based molecular diagnostics. This study used the ratio of observed to expected number of molecules and showed that it is reproducible as long as the PCR conditions are controlled. As such, the ratio of observed to expected number of molecules is indicative of the success or failure rate of a given assay on a given target molecule. The ratio should represent the primer pair efficiency for the molecule(s) targeted by the assay and, ideally, should be close to 1 (100% success rate). This primer efficiency is distinct from the amplification efficiency. Furthermore, a ratio can be established for all known targets, thus providing a benchmark for other closely related DNA molecules that may interfere in the assay and cause misprimed amplification.

These observations are consistent with the recommendations of the MIQE guidelines for proper quantitative assessment of accuracy, specificity and sensitivity. Sensitivity is defined as the limit of detection of an assay, and is well described in the MIQE guidelines [17]. Although accuracy (the difference between experimentally measured and actual concentration) and analytical specificity (detection of the appropriate target sequence rather than other sequences also present) are well defined in the MIQE guidelines, the procedures for determining these important parameters are not described. The first and most important factor influencing these two parameters is the method used to determine the number of molecules in the sample. Our results show a very strong correlation between the molecules obtained with LRE and average standard curves which indicates that these numbers should be considered as the correct number of molecules detected by an assay. The difference between the number of molecules determined by LRE (or average standard curves with perfect match primers) and the number of molecules in the sample is mostly a measure of analytical specificity and should be presented as priming probabilities for target or other nonspecific targets. Our results show that amplification profiles shift when mismatches are introduced and that standard curves can compensate for this shift because they depend on input target number of molecules during their construction. Therefore, evaluating accuracy with standard curves is appropriate because it consistently reports the number of molecules present in the sample. Standard curves should also be used for determining the limit of detection of an assay. Figure 2 provides examples of assay description with different priming probabilities. From these examples it is clear that assays can be accurate without being at their maximal specificity. However, these results also show that designing accurate assays that are less specific always decreases sensitivity.

The voluntary introduction of mismatches in primers is commonly used in combination with 3' end single nucleotide polymorphism during amplification refractory mutation system (ARMS-PCR) with the clear objective of destabilizing primer annealing [23]. This has long been thought to increase the specificity of traditional PCR diagnostic assays relying on the presence or absence of a fragment on agarose gels [23]. However, our qPCR results and those of others [24] clearly show that the introduction of mismatches shifts the C_q _towards higher values and decreases the number of molecules. Therefore, this practice decreases the analytical specificity because only a fraction of the molecules in the sample are counted. The apparent increase in specificity is unfortunately associated with a shift in the amplification profile such that no band in observed on agarose gels, or in qPCR, that the negative allele produces a C_q _above the accepted threshold for an assay. Similar results could have been obtained by reducing the amount of DNA used in the assay and using oligonucleotides without voluntary addition of mismatches. As a consequence, the introduction of additional mismatches inevitably reduces the sensitivity of assays as demonstrated in Figure 2.

A recent review of clinical applications of rapid diagnostic test methods identified PCR as the most promising technology for the detection and identification of bacterial intestinal pathogens in feces and food [25]. However, the study also reported disparate results when comparing the established culture procedures and PCR-based diagnostics, as the latter always yielded more positive results. Because of the lack of a common reference between the two technologies, it has therefore been impossible to distinguish between the lack of sensitivity of cultures and the lack of specificity of rapid testing [25]. Amplification probabilities to known specific and interfering targets that are associated with such assays would provide a means of evaluating their specificity and sensitivity and would likely help to explain some of the discrepancies between rapid testing and traditional culture-based methods. Moreover, the combination of primer pairs with established probabilities for false positives should greatly increase the confidence level during diagnostic testing. Furthermore, mispriming probabilities can also describe assay limitations regarding the detection of target molecules in a pool of closely interfering molecules. For example, an assay with a 0.001 probability of mispriming on a non-target molecule will produce the same results whether 10 target or 10,000 non-target molecules are detected. In this regard, our genotyping assay (Assay 1) provides sufficient discriminatory power to accurately genotype individuals (two possible alleles), but has limited capacity to detect rare alleles in a pool of individuals (n individuals × 2 alleles).

However, the ability to measure amplification probabilities is dependent on the quantitative nature of qPCR. This means that evaluating assay performance as outlined here is only possible for quantitative assays, as such. Therefore, the inclusion of a quantitative component could be beneficial during assay development of PCR-based molecular diagnostics, as it would likely aid in describing, improving, or validating the robustness of assays.

### SNPs and populational analysis of gene expression

The occurrence of SNPs that could interfere with PCR priming has posed a particular problem for gene expression studies in populations where genotypic variation in the target molecules is unknown. The occurrence of SNPs in human, *Drosophila *and plant transcript sequences has been estimated to be between 1/50 and 1/250 bases [4-7]. When considering only the SNPs with population frequencies above 1%, the occurrence in humans is around 1/290 bases [8]. Since a pair of oligonucleotides used in PCR-based gene expression analysis spans an average of 50 non-overlapping nucleotides, the probability that a SNP (including rare variants) falls within one of the primers can range from 20 to 100%, when analyzing a population of genetically diverse individuals. Furthermore, that same probability can be estimated to be 17% for variants with population frequencies of 1% and above in humans. This potential limitation of PCR-based assays becomes a major concern when numerous genes are analyzed. This concern was supported by a recent study using oligonucleotide microarrays that identified a dramatic lack of concordance between differential expression results analyzed with or without masking sequence variants, which affected approximately 16% of the array's probe sets [1]. The mispriming probabilities of Assay 2 and the results presented in Additional file 1 illustrated that PCR conditions can be modulated to minimize the influence of SNPs, thereby reducing the large number of aberrant measurements of gene expression that could be expected due to SNPs present in primer binding sites.

The best approach to developing PCR assays that are insensitive to SNPs is to avoid them during oligonucleotide design by using software, such as SNPmasker [26], designed for this purpose. Currently, there are over 50 million SNP submissions to consider for the human genome in build 129 of dbSNP . Twenty-seven percent of those have been added to dbSNP in the last 6 months, which indicates that periodic reevaluation of the oligonucleotide design is essential for such an approach to be viable. Furthermore, extensive knowledge of SNPs is available for very few species. Including humans, only 10 species (7 mammals, 2 insects, 1 plant) have more than one million dbSNP entries, while an additional three species have between 100,000 and one million entries in dbSNP (1 mammal, 1 fish, 1 protozoan). The dbSNP is not the only repository for SNPs and further efforts are needed to identify an appropriate genomic diversity resource. For example 0.56 million SNPs for the model plant *Arabidopsis *are available on the TAIR website (The *Arabidopsis *Information Resource) whereas there are only 301 dbSNP entries. Outside of the very few model organisms with large SNP collections, information is greatly lacking for most species that would allow researchers to effectively avoid SNPs during oligonucleotide design. For those species, our study has shown that expression assays can be designed with greatly reduced sensitivity to SNPs. Genotyping of individuals for the presence of SNPs in oligonucleotide binding sites should also be considered, especially for data validation or confirmatory analyses.

The results presented here demonstrated that the influence of SNPs can be diminished, for nucleotides ranging from the 5' terminus to the fourth nucleotide from the 3' end, in oligonucleotides that were designed at melting temperatures ranging from 62 to 66°C. The impact of SNPs situated closer to the 3' end was not tested as such, but their stronger impact is more likely to be difficult to circumvent. Consequently, the probability that a SNP influencing quantification will occur in a primer pair may be recalculated, assuming that only the last three nucleotides of each primer have an effect on qPCR; the probability would subsequently drop from 20–100% to 2.5–6%, and as low as 2% for SNPs with a population frequency of 1% or higher. The likelihood of two or more SNPs occurring was, of course, much lower.

### Current knowledge versus priming probabilities

Current knowledge regarding PCR assay design has been mostly intuitive and based on many years of optimizing PCR conditions by trial and error. For example, such intuitive knowledge would have predicted small differences in performance between Assay 1 and Assay 2 because both assays used the same primers at the same A&E temperature. However, since A&E times differed between assays, intuition would have predicted that the assay with the shortest time of A&E would have been more specific, or had greater discriminatory potential. Examination of the priming probabilities (Table 3) predicted the opposite response. The data presented in Figure 5 and Table 4 confirmed that the predictions based on priming probabilities were more accurate; suggesting that PCR conditions, including master mix composition, have great impacts on quantitative PCR. Consequently, priming probabilities are good and universal features to quantitatively assess assay performance because they can be measured with any primer pair, on any given target, with any master mix, and in any PCR conditions. Furthermore, priming probabilities are essential to describe analytical specificity as required in the MIQE guidelines [17]. These results also have underscored the importance of PCR assay conditions in the determination of assay specificity.

## Conclusion

The major challenge for designing PCR assays is to detect all molecules of interest without detecting interfering molecules. Our results have shown that mispriming, or priming, on a given target occurs with a measurable probability under standardized PCR conditions, which is broadly applicable for quantitative description of PCR assay performance. Therefore, false positive rates can be established for all known interfering molecules in molecular diagnostic applications. Similarly, priming probabilities can be used to describe the relative sensitivity to SNPs of assays designed to measure gene expression in population studies. Our results also demonstrate that although primer design is critical for successful PCR, other parameters influencing primer to template annealing are equally important for assay design. For PCR based diagnostic purposes, where power of discrimination is critical, users should favor more specific master mixes, place SNPs as close as possible to the 3' end of primers and optimize temperature, and annealing and elongation times. In contrast, for accurate transcript quantification in populations, primer design should avoid known SNPs, utilize master mixes that are less impacted by SNPs, increase the differences between primer Tm and annealing temperature and, use longer primer annealing and elongation steps.

## Authors' contributions

BB designed the experiments, performed data analysis and drafted the manuscript. ND performed the experimentation and participated in preliminary analysis. JM participated in the data analysis and manuscript preparation.

## Supplementary Material

Additional file 1 Additional file including figures and tabular data. Click here for file
